# The Resilience of the Phonological Network May Have Implications for Developmental and Acquired Disorders

**DOI:** 10.3390/brainsci13020188

**Published:** 2023-01-23

**Authors:** Michael S. Vitevitch, Nichol Castro, Gavin J. D. Mullin, Zoe Kulphongpatana

**Affiliations:** 1 Department of Psychology, University of Kansas, Lawrence, KS 66045, USA; 2 Department of Communicative Disorders and Sciences, University at Buffalo, Buffalo, NY 14260, USA

**Keywords:** network science, phonological network, resilience, aphasia, computer simulation

## Abstract

A central tenet of network science states that the structure of the network influences processing. In this study of a phonological network of English words we asked: how does damage alter the network structure (Study 1)? How does the damaged structure influence lexical processing (Study 2)? How does the structure of the intact network “protect” processing with a less efficient algorithm (Study 3)? In Study 1, connections in the network were randomly removed to increasingly damage the network. Various measures showed the network remained well-connected (i.e., it is resilient to damage) until ~90% of the connections were removed. In Study 2, computer simulations examined the retrieval of a set of words. The performance of the model was positively correlated with naming accuracy by people with aphasia (PWA) on the Philadelphia Naming Test (PNT) across four types of aphasia. In Study 3, we demonstrated another way to model developmental or acquired disorders by manipulating how efficiently activation spread through the network. We found that the structure of the network “protects” word retrieval despite decreases in processing efficiency; words that are relatively easy to retrieve with efficient transmission of priming remain relatively easy to retrieve with less efficient transmission of priming. Cognitive network science and computer simulations may provide insight to a wide range of speech, language, hearing, and cognitive disorders.

## 1. Introduction

**Figure 1:**
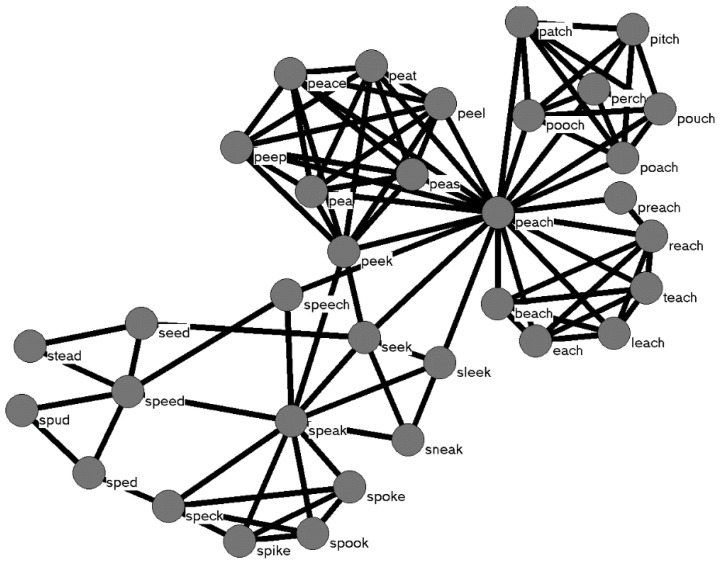
Nodes represent words, and connections are placed between words that sound similar to each other. In this example, phonological similarity is defined by a simple computational metric (add, delete, or substitute a phoneme in a word to form another word), but phonological similarity can be defined in other ways [7,8].

Network science is being used increasingly in a wide range of disciplines, including the cognitive and language sciences [1,2,3,4,5]. In this approach, *nodes* represent some entity in a system, such as words in the mental lexicon, and *connections* are placed between nodes that are related in some way, such as words that are phonologically [3] or semantically related [6]. In a system with many nodes and connections, a web-like *network* emerges, like the network in Figure 1 for the word *speech* and other words that are phonologically similar to it.

In [3], the structure of a network of English words was examined with connections placed between similar sounding words (as in Figure 1). It was found in [3] that the network had a number of features that were not observed in networks of social or technological systems, nor in networks of words where connections were placed between semantically related words. To examine whether the constellation of network features observed in [3] was unique to English or might be found in other languages as well, the phonological networks of words in Spanish, Mandarin, Hawaiian, and Basque, which were selected to be representative examples of different language families, were examined [9]. Although English and Spanish are both from the Indo-European family of languages, English is a Germanic language, whereas Spanish is a Romance language. Mandarin is a Sino-Tibetan language, Hawaiian is an Austronesian language, and Basque (or Euskara) is a linguistic isolate, meaning that it has not (yet) been identified as a member of a given language family. Despite these five languages differing from each other in their morphology, phonemic inventories, typical word-length, canonical syllable shape, use of tone, etc., it was found by [9] that all five languages had network structures that were similar to the phonological network of English first observed in [3].

A central tenet of network science is that the structure of the network influences processing in that system [10]. For example, networks with a small-world structure have a comparatively short average path length between nodes in the system, and the nodes are highly interconnected, which makes searches through such networks more efficient than searches using the same search-algorithm in a network organized in a different way [11,12]. The unique constellation of features in the phonological network motivated subsequent research using methods from psycholinguistics and cognitive psychology (for a review see [5]) to examine how a number of network measures might influence the cognitive processes involved in spoken word recognition [13], speech production [14], word-learning [15], long- and short-term memory [16], and the perception of the speech to song illusion [17]. Influences of certain network measures on processing have not only been observed in typically developing language users, but have also been observed in people who stutter [18] and in people with aphasia [19].

For a period of time, this constellation of network features was thought to be unique to phonological networks (see also [20]). However, an analysis of the DarkNet [21]—sort of a secret version of the Internet where anonymous users often engage in illegal activities—discovered that the DarkNet, like the phonological network, also lacked a large core of highly connected nodes. Given that the structure of a network influences processing in that system [10]—regardless of what system the network represents—the discovery of another system with network structures similar to the phonological network provided a unique opportunity to learn more about and to corroborate findings in the phonological network. The lack of a large core of highly connected nodes in the phonological networks and in the DarkNet has some interesting implications. For example, when [21] simulated targeted attacks and random failures on the network structure of the DarkNet they found that the DarkNet was much more resilient to failure than the different network structure found in the Internet [22]. Similarly, it was found that phonological networks were also more resilient to targeted attacks and random failures, suggesting that this network structure may not only contribute to the difficulty in dismantling the DarkNet (which would interfere with the illegal activities engaged upon it), but also in the resilience of various language processes despite the damage caused by aging, stroke, disease, or disorder [9].

Given the clear influence that the structure of the phonological network has on various language processes we wondered how damage to the phonological network might affect processing. Previous studies have examined a similar question regarding the resilience of semantic networks (this concept is referred to as percolation theory in the network science literature) looking at semantic networks that change due to typical aging [23,24], Alzheimer’s disease [25], aphasia [26], and other clinical conditions [27]. Percolation analysis has also been used to examine the semantic networks of people varying in their level of creativity [28], and in languages other than English (e.g., Hebrew [29]).

In the present study, we focused on the phonological network of English words, and asked: how does the continued and random removal of connections alter the overall structure of the phonological network (Study 1)? How might that damaged structure of the phonological network influence processing (Study 2)? Finally, how might the structure of the intact network “protect” processing even when a less efficient algorithm is used (Study 3)? The last research question was motivated by the work of [11,12], who examined how the same search algorithm performed on networks with different structures. Instead, we examined how different algorithms might perform on the same network to determine if certain network features conferred some protection to phonological processing.

## 2. Study 1: Damaging the Phonological Network

The structure of the phonological networks of English, Spanish, Mandarin, Hawaiian, and Basque were examined in [9] and it was found that all five languages had similar phonological network structures. Given the unique structure of the phonological networks observed in [9], which differed from the structure typically seen in networks of social or technological systems, the researchers decided to evaluate the resilience of the networks. That is, how well do these unique networks stay connected despite being damaged?

Networks can be damaged by removing nodes (and their associated connections), or by removing just the connections. The results produced by these two approaches are similar. Further, the removal of the nodes/connections can be carried out at random or by targeting nodes with many connections (i.e., in order of degree). One way to assess resilience in a network is by looking for changes in the *average shortest path length*, which measures how many connections must be traversed on average to get from one node to another in the network and quantifies how easy it is to transmit information between any two nodes in the network [9]. In networks with the scale-free structure typically seen in social or technological systems, the network tends to remain well connected as indicated by the average shortest path length remaining constant when nodes are randomly removed. However, when nodes are removed in order of degree, the average shortest path length increases dramatically [30], indicating that part of the network has fractured, making it more difficult to reach certain nodes (i.e., a longer path must now be taken to get from A to B).

In the phonological networks examined in [9], up to 5% of the nodes in the network (about 1000 nodes) were removed, and across that range of damage the networks remained well connected (i.e., the average shortest path length remained constant). This was true whether node removal was conducted at random or by targeting highly connected nodes, which contrasts with the pattern typically observed in social and technological systems with scale-free network structures. Recall that in social and technological systems, such systems remain well connected when damaged at random, but “fall apart” when nodes are removed in the order of degree (i.e., targeting highly connected nodes). The researchers suggested that the resilience of the phonological networks may contribute to the resilience of language processing despite injury to or degradation of the language-related areas of the brain.

In the present study, we wished to examine further the resilience of the phonological network by analyzing the phonological network over a wider range of damage than had been examined in [9]. Recall that in [9] up to 5% of the nodes in the network were removed in their analysis. In the present study, we damaged the network more extensively by randomly removing up to 90% of the connections in the network. 

In further contrast to the work by [9], we removed connections instead of nodes from the network. Although the results are typically similar regardless of whether nodes or connections are removed, we reasoned that removing connections was a closer analogue to the difficulties sometimes faced in lexical retrieval in typical speakers and in individuals with developmental or acquired language disorders [31]. For example, in the tip of the tongue state the speaker might be able to retrieve some information from the lexicon related to the meaning, gender, or syntactic class of a word [32], but they are not able to completely access the phonological word-form, perhaps retrieving the number of syllables the word has, or the sound with which it starts [33]. Note that the word is not unlearned or forgotten forever. Indeed, the tip of the tongue state might be resolved spontaneously later in the day, or by consulting a dictionary or other resource. By damaging the network in this way, the words/nodes are still in the lexicon, but the damage to the network structure may make lexical retrieval more difficult by creating a longer path that must be traversed in order to retrieve the word/node.

Finally, we used only the random removal of connections to damage the network. We did not target connections in some way as one might when removing nodes (i.e., in the order of degree, k-core, etc.).

### 2.1. Methods

We started with the phonological network examined by [3], which contains 19,340 nodes (representing words) in the network. A connection was placed between two words/nodes if the addition, deletion, or substitution of a single phoneme changed one word into the other. A subset of this network is represented in Figure 1. The “intact” network contained 31,267 connections. We then randomly removed 10% of those connections to form a new network.

From the network with 10% damage, we randomly removed another 20% of the connections resulting in a network that (compared to the intact network) had 30% of the connections removed. We continued to randomly remove connections using the previous network as the starting point to produce networks that had 50%, 70%, and 90% of the connections (compared to the intact network) removed to produce networks with varying amounts of damage. We used this method of damaging the network so that we could examine a word in its “intact” state and then follow the processing of that word (and its connections) longitudinally (see Study 2 below). Other methods of damaging the network, such as randomly removing a percentage of the intact network and then resetting the network to remove a different percentage of the intact network would not allow us to examine specific words longitudinally.

Further, Ref. [34] compared strategies to attack networks such that nodes/connections were targeted based on their “importance” (i.e., measures of degree and betweenness centrality) in the initial network or based on measures of their “importance” that were recalculated in the damaged network. They found that continuing to recalculate “importance” on the damaged network led to greater damage in subsequent attacks compared to when “importance” continued to be based on the initial network structure. The results of [34] therefore suggest that the changes that occur in the network as a result of attack are important to take into account for subsequent attacks. Although we are not making targeted attacks on the phonological network, we considered the impact that the current (damaged) network structure might have on forming the subsequent network structure (with increased damage) when deciding how to damage the phonological network in the present study.

We used Gephi (version 0.9.2) to measure various structural features of the resulting networks. *Giant component size* refers to the largest group of connected nodes in a network. In addition to reporting the number of nodes in the giant component, we also report the percentage of nodes in the network found in the giant component. *Lexical islands* (typically called just *components* in the network science literature) are groups of words that are connected to each other, but not connected to the giant component. The *number of isolates* (sometimes referred to as “lexical hermits”) refers to the number of nodes with degree = 0; that is, the number of words that are not connected to any other word. In addition to reporting the number of isolates, we also report the percentage of nodes in the network that are isolates.

The *number of communities* (also referred to as *modules*) refers to the number of sub-groups of nodes within the giant component that are more connected to each other than they are to nodes outside of the community [35,36]. The *modularity value* (*Q*), measures the extent to which clear, well-defined communities are found in a network (Ref. [37] see [35] for a formal definition). Positive values of *Q* close to the maximum of +1.0 indicate the presence of clear, well-defined communities in the network.

*Average degree* is the mean number of connections per node. We report this value for the entire network (which would also include nodes in the lexical islands and the lexical hermits in that calculation), and just for nodes in the giant component.

*Average clustering coefficient* is the mean clustering coefficient, which measures the proportion of phonological neighbors of a word that are also neighbors of each other (see [13] and others for a more formal definition of clustering coefficient). We report this value only for nodes in the giant component.

*Average shortest path length* (ASPL) is the mean distance (measured as the number of connections) between any two nodes in the network (restricted to the giant component). ASPL will serve as an indication of how resilient the phonological network is with increasing amounts of damage. A resilient network will have a constant ASPL across increasing amounts of damage, whereas a network that “falls apart” will show an increase in ASPL with increasing amounts of damage.

### 2.2. Results

**Figure 2:**
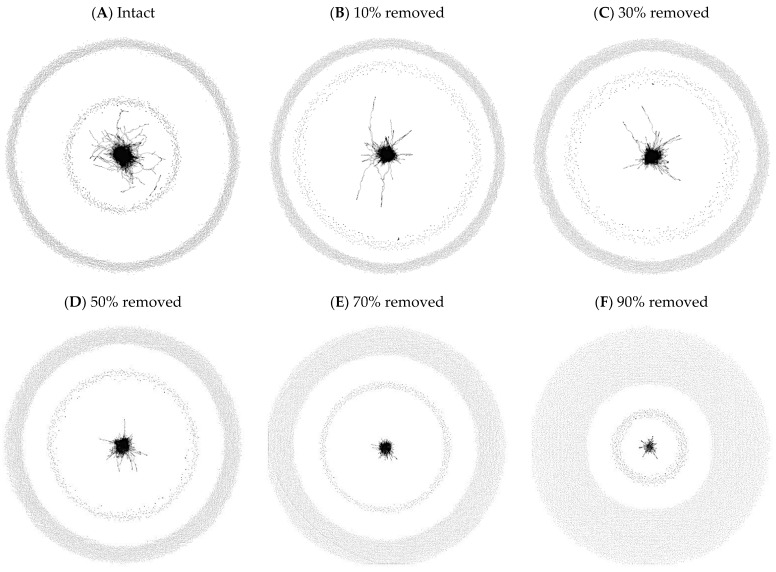
Panel (**A**) shows the intact network. Panels (**B**–**F**) show the damaged phonological networks. Each network contained the same 19,340 words (i.e., nodes), but increasing percentages of connections between words were randomly removed. The giant component is shown in the center of each network. The inner ring contains the smaller components (sometimes called “lexical islands”), and the outer ring contains the isolates (sometimes called “lexical hermits”).

Table 1 shows the *number of connections* in the intact and damaged networks. Further summarized in the table are the giant component size (% of the network in GC), the number of communities and modularity value, average degree for the entire network and just for the nodes in the giant component, average clustering coefficient, and average shortest path length (ASPL) for each of the networks. Note that the values reported in Table 1 are “points” not mean values with distributions. There is no way to test for the statistical difference between point values, because there are not enough degrees of freedom for any statistic to use. Given the importance of ASPL in assessing the resilience of the network we therefore used the informal heuristic that the ASPL must be greater than or approximately equal to 1.5 times the value of the intact network to be considered “significantly” different (ASPL in intact network = 6.05 × 1.5 = 9.075). A similar heuristic was used in [10] when assessing differences in path length. Using this heuristic, only the ASPL for the network with 90% damage differed significantly from the ASPL of the intact network. Visualizations of the intact and five damaged networks can be found in Figure 2.

### 2.3. Discussion of Study 1

In the present study, an increasing number of connections were removed from an intact network in order to investigate the resilience of the phonological network. That is, how much damage can the network sustain before it is no longer well-connected and effectively “falls apart”? In contrast to a similar analysis in [9] where up to 5% of the nodes were removed (at random and in a targeted fashion) from the network to assess resilience, in the present study we instead randomly removed connections between nodes, and did so over a wider range of “damage” to the system (up to 90% of the connections were randomly removed). Because words are not unlearned or forgotten, we reasoned that removing connections rather than removing nodes was a closer analogue to certain types of retrieval problems (e.g., the tip of the tongue state, developmental or acquired language disorders). Recall that [9] observed that over the range of damage they inflicted on the phonological network, the system was quite resilient. That is, the network remained relatively intact as indicated by the average shortest path length remaining relatively constant for both random and targeted removal of nodes.

In the case of the phonological network in the present study, we also see the average shortest path length (ASPL) in the giant component remain fairly constant (at a value ~6 connections) until 90% of the connections in the network were removed resulting in the average shortest path length nearly doubling (from ~6 to ~12 connections). The marked increase in the average shortest path length is indicative that the significantly damaged networks are less interconnected than the intact and less-damaged networks, and are effectively being “pulled apart”. With less interconnectivity, there are fewer “short-cuts” through the network to aid in the rapid traversal of the system, forcing one to take the “long way” from node A to node B. It is remarkable that the network remained resilient (i.e., well connected) until more than half of the connections were removed from the system.

A number of other network measures support the conclusion that the significantly damaged networks are less interconnected than the intact and less-damaged networks, and have been effectively “pulled apart”. Consider the decrease in the size of the giant component and the increase in the number of isolates (i.e., “lexical hermits”) as an increasing number of connections are removed from the phonological network. The only place that still has many connections is the giant component, making it likely that a randomly removed connection will be removed from a node in the giant component. With increasing amounts of damage those nodes end up being stripped of all connections to the giant component, and are cut off to become an isolate (or part of a very small lexical island/component).

**Figure 3:**
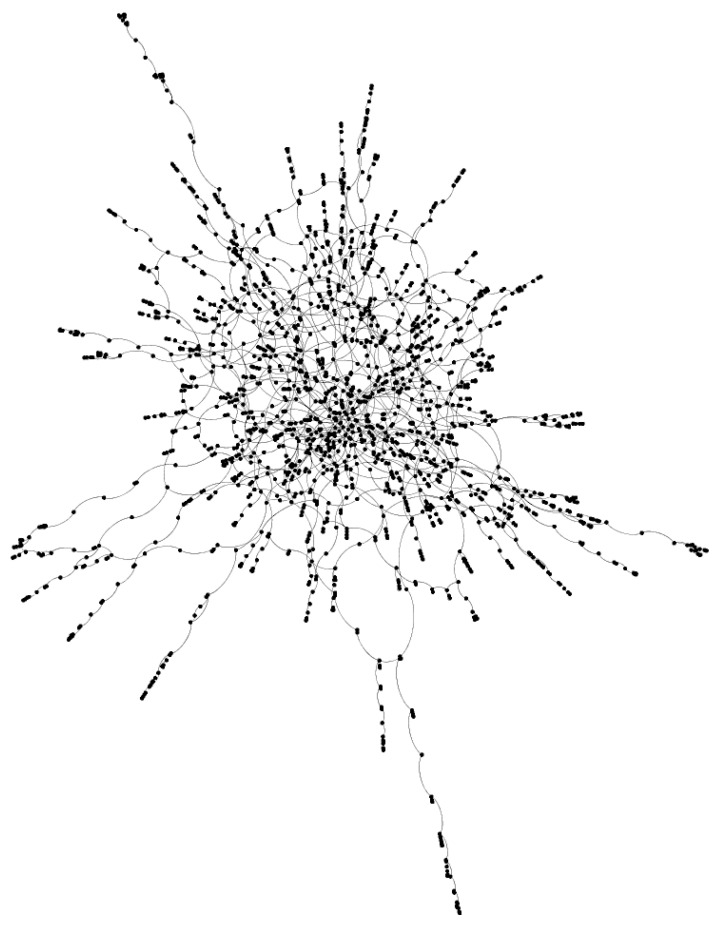
The giant component of the phonological network with 90% of the connections removed.

The increase in the number of communities in the giant component also suggests that the highly interconnected giant component is getting stretched and pulled apart, forming increasingly smaller communities that are only weakly connected to each other in the giant component. Figure 3 shows the giant component of the network with 90% damage, which resembles pearls on a string more than the highly interconnected web-like structure typically evoked by the word network. Indeed, the decrease in the average degree and average clustering coefficient of nodes in the giant component further attests to nodes in the giant component being “strung out” and less interconnected by the increasing amounts of damage inflicted on the network. See the work in [38] on theoretical networks for the influence of targeting for removal nodes that connect communities/modules on the resilience of the network.

Recall that a central tenet of network science is that the structure of the system influences processing in that system [10]. Given the changes in the structure of the network that we observed when connections between nodes were randomly removed, we used in Study 2 computer simulations on the damaged networks to examine how the (damage to the) structure of the phonological network might influence processing.

## 3. Study 2: Simulated Language Processing on the Damaged Phonological Networks

Because it is unethical to harm human participants in order to mimic the damage that might occur in certain types of developmental or acquired language disorders, we turned to computer simulations to explore how language processing might be affected by the changes we observed in Study 1 in the structure of the phonological network as a function of increasing removal of connections (e.g., [39]). Although the phonological networks in Study 1 only capture the structural relationship among words in the mental lexicon, processing in these structural models can be modeled by either a random walk (e.g., [40]) or the diffusion of activation—akin to spreading activation—across the network (e.g., [41]). Similar computer simulations were used in [13,41,42,43] to explore typical language processing in the phonological network.

In [43], an *R* package called *spreadr* is described that can diffuse activation across a network provided by the user over a range of time-steps, initial activation levels, etc. We used *spreadr* (version 0.1.0) in the present study to diffuse activation across the damaged phonological networks from Study 1, allowing us to examine how damage to the structure of the network might influence processing. We examined in two ways how damage to the structure of the network might influence processing: (1) by examining how the spread of activation from a word changed with increasing amounts of damage; and (2) by comparing the performance of the network to the performance of humans in a language processing task for a subset of words in the network.

The subset of words we used in the simulation came from the Philadelphia Naming Test (PNT; [44]); more details about these words are provided in the Methods section. We compared the performance of the damaged network for the words in the PNT to performance on the PNT of adults who acquired certain types of aphasia. The decision to use performance data from people with aphasia (PWA) was made in part out of convenient access to the data. In part, we also used these performance data because damage to a phonological network seemed like the most direct analogue to the described access deficit we modeled in Study 1 [31]. Note, however, that we believe our findings apply to other types of developmental and acquired language disorders regardless of the underlying factor that causes damage to the network (see also Study 3).

As background, aphasia is an acquired language disorder that often occurs after a stroke and results in impaired comprehension and/or production of language. According to [45], approximately 1/3 of strokes result in aphasia (approximately 225,000 people) and there are currently at least 2,000,000 people in the USA living with aphasia [46]. The classic typology of aphasia has resulted in broad diagnostic categories including: Broca’s Aphasia (hallmarked by impaired fluency and repetition), Wernicke’s Aphasia (hallmarked by impaired comprehension and repetition), Conduction Aphasia (hallmarked by impaired repetition), and Anomic Aphasia (hallmarked by impairment in naming).

Impaired word retrieval is a ubiquitous symptom of aphasia, leading to a primary focus on word retrieval in many aphasia treatments. Across aphasia types, word retrieval impairments can stem from impaired phonological knowledge caused by left perisylvian damage [47], impaired semantic knowledge caused by damage to association cortices in dominant and nondominant hemispheres [48], or impairment in connecting semantics and phonology caused by damage to white matter connections between perisylvian regions and association cortices [49]. Since the predominant types of aphasia (e.g., Broca’s, Wernicke’s, and Conduction) tend to result from left perisylvian damage or connections to left perisylvian regions, some degree of phonological impairment is found in most people with aphasia, justifying our use of a phonological (but not a semantic) network in the present work (cf., [26]).

To examine how increasing amounts of damage to the network influences processing we compared the activation levels of each word across different levels of damage in the phonological network. We made the intuitive prediction that activation levels of the words will decrease with increasing amounts of damage to the network. This change in activation levels with damage reflects the increased difficulty of accessing the words from the lexicon due to increasing amounts of damage to the network.

We also predicted that stimulus words with larger activation values would correlate with higher accuracy and fewer errors by PWA. To test our prediction, we conducted Pearson correlations for each aphasia type between the activation value of stimulus words after 5 timesteps from the network with 90% of the connections removed and the average accuracy of PWA for the stimulus words. We focused on the activation values from the network with 90% damage because, as shown in Table 1, that was the network that exhibited a significant increase in the average shortest path length, suggesting that the connectivity of the network had been significantly disrupted.

### 3.1. Methods

The lexicon in the present simulation consisted of the 19,340 English words in the phonological network examined in [3], and also used in Study 1. The stimulus words that were presented to the network to “retrieve from the lexicon” consisted of 165 words from the 175 stimulus words in the Philadelphia Naming Test [44] that were found in the lexicon used in the simulation (see Appendix A).

#### 3.1.1. Spreadr

We used the following parameter settings in *spreadr* (version 0.1.0) for the simulation reported here. With this combination of parameters, we conducted a simulation of spreading activation on each of the damaged phonological networks reported in Study 1 (10%, 30%, 50%, 70%, and 90% damaged networks).

An *initial activation* value of 20 units was used for each stimulus word in the present simulation. Although activation = 100 units in the simulations reported in [41], this value is arbitrary. A smaller value was selected in the present simulations to reduce computational burden, thereby accelerating data collection.

*Decay* (*d*) refers to the proportion of activation lost at each time step. This parameter ranges from 0 to 1, and was set to 0 in the simulations reported here to be consistent with the parameter settings used in [41,42].

*Retention* (*r*) refers to the proportion of activation retained in a given node after it diffused activation to other nodes connected to it. This value ranges from 0 to 1, and was set to 0.5 in the simulations reported here. In [41] values ranged from 0.1 to 0.9 in increments of 0.1. Because the various retention values in [41] produced comparable results across retention values, we selected in the present simulations a single, mid-range value (0.5) for the retention parameter in order to reduce the computational burden, thereby accelerating data collection.

The *suppress* (*s*) parameter in *spreadr* will force nodes with activation values lower than the selected value to activation = 0. It was suggested in [43] that when this parameter is used a very small value (e.g., *s* < 0.001) should be used. In the present simulations *suppress* = 0 in order to be consistent with the parameter settings used in [41,42].

*Time* (*t*) refers to the number of time steps that activation diffuses or spreads across the network. In Vitevitch et al. (2011) *t* = 10, however in the present simulations *t* = 5. A smaller value was selected in the present case because as shown in Figure 3 of [43], activation values reach asymptote at approximately five time-steps. Furthermore, as shown in the hop-plot in Figure 2 of [50] approximately 50% of the network has been reached by traversing on average five connections (i.e., hops) in every direction from a given node, suggesting that the network has been sufficiently saturated. We selected in the present simulations a smaller value (*t* = 5) for the time parameter in order to reduce the computational burden, thereby accelerating data collection.

At the end of five timesteps, we documented the activation level of each of the 165 PNT stimulus words in each of the damaged networks. Larger activation values in the simulation are hypothesized to correspond to better performance in behavioral tasks (e.g., faster reaction times, more accurate responses, etc., Ref. [41]). Although some computer simulations may use relative activation of the target word compared to competing words (note that another model of language processing, Node Structure Theory, uses absolute activation of a node, Ref. [33]), in the case of *spreadr*, the dynamics of the model are such that initial activation of the target word results in the target word always being relatively more active than competing words even after activation has dispersed through the network, making the absolute activation of the target word the appropriate metric to use in the present simulations.

Further, previous simulations [13,41,42,43] typically compared two (or more) categories of target words (e.g., words that were high vs. low in clustering coefficient), which again made the absolute activation of the target word the appropriate metric to use. In the present study, we will track absolute activation in the same word across different levels of damage, and compare absolute activation in words to performance by PWA in a common psycholinguistic task, which again, makes absolute activation the appropriate metric to use. The performance data for PWA were obtained from the Moss Aphasia Psycholinguistics Project Database (MAPPD).

#### 3.1.2. MAPPD Database

We obtained PWA performance, specifically the average percentage of correct productions, on the 165 PNT words from the Moss Aphasia Psycholinguistics Project Database (https://www.mappd.org/ accessed on 8 February 2021) [51]. We further grouped the data by type of aphasia, focusing on Anomic (*n* = 181), Broca’s (*n* = 85), Conduction (*n* = 95), and Wernicke’s (*n* = 82) aphasia given their likelihood of phonological impairment and because these types of aphasia had larger sample sizes (all other *n*s < 15).

### 3.2. Results

#### 3.2.1. Analysis of Words

**Figure 4:**
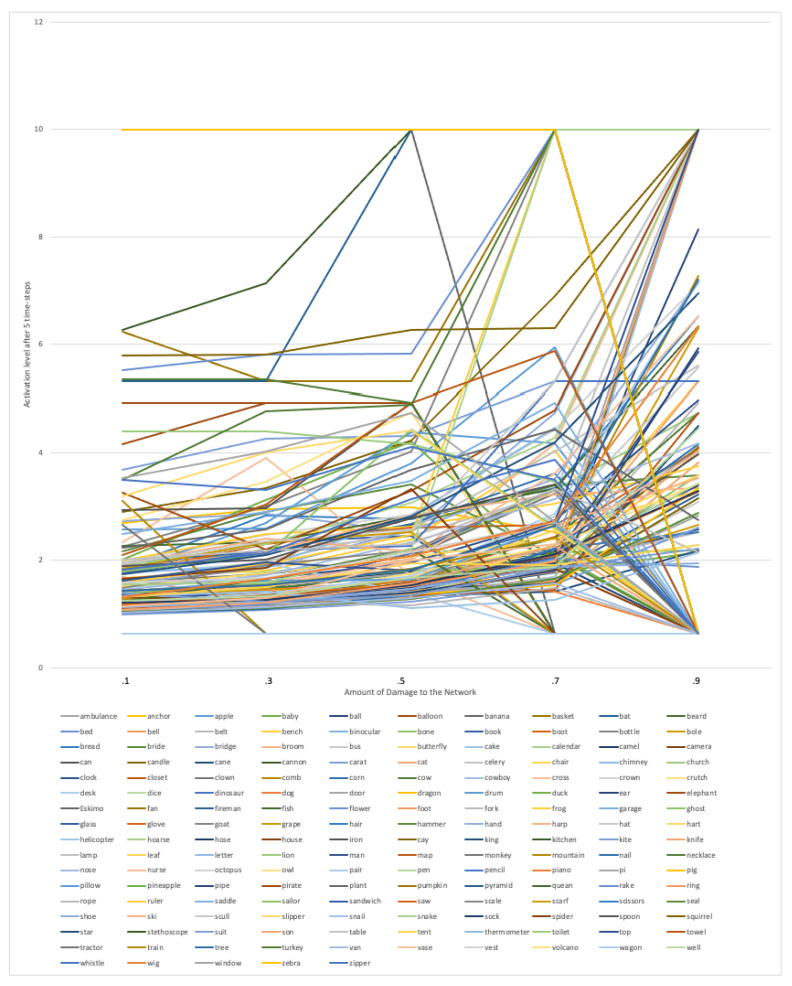
The activation level after five time-steps for each word from the PNT at each level of damage to the phonological network.

**Figure 5:**
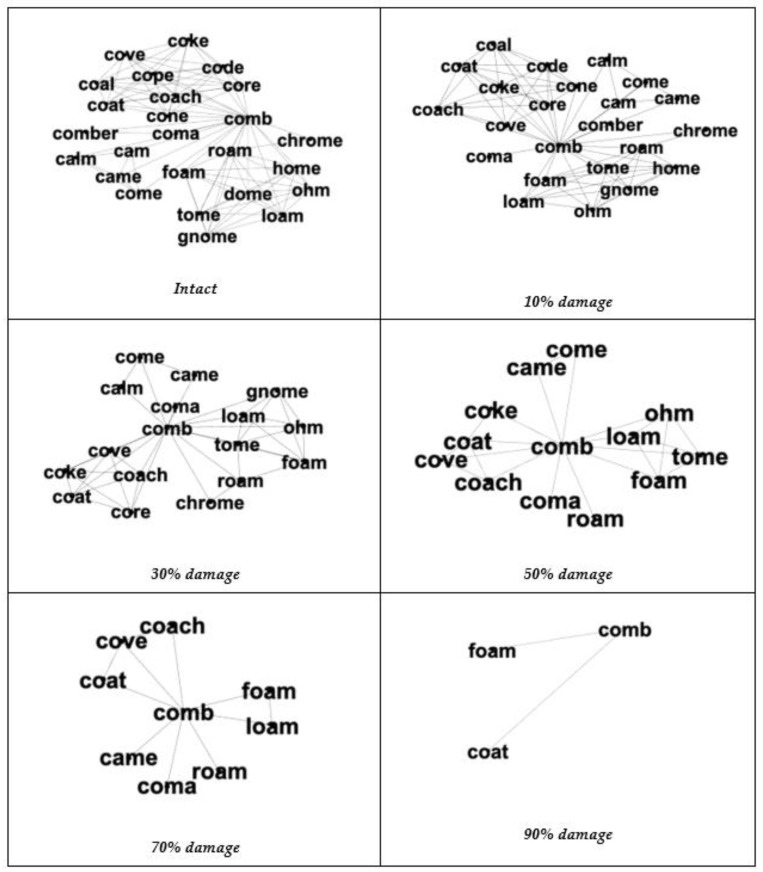
The phonological neighborhood for the word *comb* in the intact network, and in the networks with varying amounts of damage. The word *comb* is an example of a word on the PNT that started out with lower levels of activation remaining in the node after five time-steps, but increasing amounts of damage resulted in higher amounts of activation remaining in the node after five time-steps.

Figure 4 shows the activation level after five time-steps for each word from the PNT at each level of damage to the phonological network. Although the image is visually dense, we observed several patterns in the figure related to how damage to the network influenced the amount of activation remaining in the node after five time-steps: (1) a subset of nodes (25 nodes) started out as “lexical hermits” (i.e., a final activation level of 0.625) and remained lexical hermits (because connections, not nodes were removed); (2) a subset of nodes started out with higher levels of activation remaining in the node after five time-steps, but at some point the removal of connections turned the node into a “lexical hermit” (i.e., a final activation level of 0.625; 53 nodes) or a lexical island of size 2 (i.e., a final activation level of 10; 18 nodes); and(3) a subset of nodes (69 nodes) started out with lower levels of activation remaining in the node after five time-steps (at 0.1 damage), but increasing amounts of damage resulted in higher amounts of activation remaining in the node after five time-steps (at 0.9 damage). See Figure 5 for an example of the word *comb*, which started out with lower levels of activation remaining in the node after five time-steps, but increasing amounts of damage resulted in higher amounts of activation remaining in the node after five time-steps.

#### 3.2.2. Correlation of Activation Levels to PWA

The Pearson correlations between the activation value of stimulus words after five timesteps in the network with 90% damage and the average accuracy of PWA for the stimulus words showed significant positive correlations (see Table 2). Across aphasia types, the positive *r* values, ranging from 0.169 to 0.202, indicate that stimulus words with greater activation values in the simulation also tended to be more accurately produced by people with aphasia (all *p*s < 0.05). Importantly, the Pearson correlations between the activation value of stimulus words after five timesteps in the intact network and the average accuracy of PWA for the stimulus words showed no correlation (see Table 2), suggesting that the damaged network captures behavior related to developmental and acquired disorders.

### 3.3. Discussion of Study 2

In the present study we simulated lexical retrieval using *spreadr* on the damaged networks from Study 1. To assess how the damaged structure of the network might influence processing (as measured by the final activation level of the words), we compared the final activation levels of the words across networks with increased amounts of damage. We also compared the simulation results from the network with 90% of the connections removed to behavioral data (i.e., PNT naming accuracy) from persons with aphasia (PWA).

#### 3.3.1. Analysis of Words

The analysis of the final activation levels of the words across networks with increased amounts of damage revealed that our initial prediction was too simplistic. We intuitively predicted that activation levels of the words would decrease with increasing amounts of damage to the network, because damage to the network would make it more difficult to access the words. The results were more complicated than our initial prediction. Instead, we found several subsets of words with different patterns of activation across the networks.

One subset of words started out with higher levels of activation remaining in the node after five time-steps, but at some point, the removal of connections turned the node into a “lexical hermit” or a lexical island of size 2. This resulted in the word nodes having less activation as the network became more and more damaged. This subset of words was indeed in line with our initial prediction.

What we had not initially considered was that some words would start out as lexical hermits. As reported in [3], the phonological network of 19,340 words contained 10,265 lexical hermits, 2567 words occupying lexical island of various sizes, and the remaining 6508 words were found in the giant component. Given the large percentage of words in the phonological network that are lexical hermits (53%), we should have anticipated that some of the words on the PNT would be lexical hermits. We indeed found a subset of words that were lexical hermits, and whose final activation levels remained the same with increasing amounts of damage to the network. Recall that we used the approach of removing connections in the present study in order to examine changes in processing with increasing amount of damage. Had we used the approach of removing nodes instead of connections from the network, some of these word nodes may have been removed from the network at some point.

The last subset of nodes that we observed (and had not expected) consisted of words that started out with lower levels of activation remaining in the node in the network with small amounts of damage (10% of the connections removed), but higher amounts of activation when the network was significantly damaged (90% of the connections removed). An inspection of this subset of nodes revealed that these words initially had “dense phonological neighborhoods”. That is, these words were connected to many similar sounding words (or in network science terms, these nodes had high degree). Being connected to many other nodes results in the target node having a small amount of activation remaining after five time-steps.

Damage to the network essentially “pruned” some of neighbors in the phonological neighborhood of this subset of words, turning the previously dense neighborhood into a sparse neighborhood. With fewer nodes being connected to the target word, more activation remains with the target word after five time-steps. Higher levels of activation indicate that the word should be retrieved more quickly and more accurately. Indeed, many behavioral studies (for reviews see [7,52]) show that words with sparse neighborhoods (i.e., low degree, or few competitors) are responded to more quickly and accurately than words with dense neighborhoods (i.e., high degree, or many competitors). Computer simulations on phonological network models also show this [41]. Similarly, one theory for why older adults experience memory declines compared to younger adults is that older adults have richer/more cluttered representations in memory than younger adults [53]. This finding counterintuitively suggests that selected damage to the system could be beneficial.

The paradoxical benefit of improving performance by damaging the system, or pruning specific connections is not without precedent. In [25] a semantic network with weighted connections between concepts was examined and damaged to simulate how processing changed as Alzheimer’s disease progressed. They found that removing weak connections (i.e., with low weights) resulted in other connections being strengthened as the weight from the pruned connection was redistributed to the remaining connections. The strengthening through pruning that was observed in [25] in a weighted semantic network is similar to the pruning of (unweighted) connections in the phonological network increasing activation levels of target words by turning their initially dense phonological neighborhoods into sparse phonological neighborhoods.

#### 3.3.2. Correlation of Activation Levels to PWA

Using Pearson correlations, we found that the accuracy of correct picture naming by PWA correlated with activation values of stimulus words in the 90% damaged network. As predicted, stimulus words with more activation after five timesteps were also more likely to be produced correctly by PWA, aligning with models of spreading activation and word retrieval (e.g., [54,55]).

The results of the correlation analyses in the present study complement the regression analysis reported in [19], where network measures of words in the intact phonological network were used to predict the naming accuracy of items in the Philadelphia Naming Test for age-matched controls, individuals with Broca’s Aphasia, and individuals with Wernicke’s aphasia. Focusing just on the network measures that [19] reported, they found that *degree* (known as *phonological neighborhood density* in the psycholinguistic literature) influenced naming accuracy such that words with high degree/many phonological neighbors were named more accurately than words with low degree/few phonological neighbors, replicating previous findings in studies of speech production in healthy adults (e.g., [56,57,58,59,60]). It was also found by [19] that the location of a word in the network—in or outside of the giant component (either in an island or an isolate)—influenced naming accuracy. They observed that words found outside the giant component were named more accurately than words in the giant component (see also [61]).

Given the findings of the correlation analysis in the present study, and the previous findings of [19] demonstrating that the structure of the phonological network influences naming performance in the PNT, we conducted a post hoc analysis to further examine the phonological errors in the MAPPD database for additional evidence that the structure of the (damaged) phonological network influences processing. In this analysis, we considered the target words that were erroneously produced as either formal errors (F) or nonwords (N) (but not Semantic (S) errors, Visual errors, etc.) in the MAPPD database that were made by those individuals classified (based on the score on the PNT) as having mild (*mean* = 85.62; *sd* = 5.25; *n* = 1569 errors) or very severe anomia (*mean* = 25.2; *sd* = 0.0; *n* = 43 errors). To be clear, we are not analyzing the formal errors, nor the nonwords that were produced, but instead looked at the characteristics of the intended (target) word. Indeed, nonwords do not exist in the network we used in the present simulation, making it difficult in the present study to analyze the actual nonword productions. Note, however, that [62] examined other network architectures that would be able to produce nonwords.

For each formal/nonword error we examined the location in the network (in or outside the giant component) for the target word. Recall that [19] found that words outside of the giant component were named more accurately than words in the giant component. This may seem counterintuitive because words in the giant component tend to be short in length, monosyllabic, acquired early in life, and occur frequently in the language, etc., which are all characteristics that typically result in faster and more accurate language processing [61]. Furthermore, if one randomly selected a word from the lexicon to make an error on, it is more likely to be a word that is outside of the giant component, where most of the words in the phonological network are located. Thus, if the location of the word in the (damaged) phonological network influences processing, then, based on [19], we should see more errors made on target words found in the giant component than on target words found outside of the giant component.

We used the location of the word in the network with 10% of the connections removed for the 1438 formal/nonword errors made by people with mild anomia that were found in the phonological network. As shown in Table 1, the network with 10% of the connections removed has 33% of the words in the giant component, and 67% of the words outside of the giant component (in smaller components or as isolates). The people with mild anomia made 56% of the formal/nonword errors on target words that are located in the giant component, and 44% of the formal/nonword errors on target words that are located outside of the giant component. A chi-square test to compare the actual error rates to the expected error rates (based on random selection from where words are located in the damaged network) shows that there is a significant difference (χ^2^ = 10.71, *p* < 0.01). More errors were made on words in the giant component (and fewer errors for words outside of the giant component) than one would expect if you randomly selected a word from the network, as we predicted based on the findings of [19].

We used the location of the word in the network with 90% of the connections removed for the 42 formal/nonword errors made by people with very severe anomia (n.b., all of these words were found in the phonological network). As shown in Table 1, the network with 90% of the connections removed has 9% of the words in the giant component, and 91% of the words outside of the giant component (in smaller components or as isolates). The people with very severe anomia made 35% of the formal/nonword errors on target words that are located in the giant component, and 64% of the formal/nonword errors on target words that are located outside of the giant component. A chi-square test to compare the actual error rates to the expected error rates (based on random selection from where words are located in the damaged network) shows that there is a significant difference (χ^2^ = 20.90, *p* < 0.00001). Again, more errors were made on words in the giant component (and fewer errors for words outside of the giant component) than one would expect if you randomly selected a word from the damaged network, as we predicted based on the findings of [19]. Therefore, evidence from the naming accuracy rates and words that were produced erroneously both suggest that the structure of the (damaged) network may influence lexical processing (as measured by the activation levels in the network simulation).

## 4. Study 3: Simulated Language Processing with an Algorithm Varying in Efficiency

In Study 1 we examined how damage to the phonological network affected the overall structure and resilience of the network. In Study 2 we used computer simulation and analyses of performance on a naming task by PWA to examine how the structure of the damaged phonological network influenced processing. In the present study we again used computer simulation, but this time instead of damaging the phonological network we “damaged” the processing mechanism to examine if the structure of the network might confer some protection to cognitive processes when the processing algorithm becomes less efficient. This investigation can be viewed as the inverse of work by [11,12], who examined how the efficiency of the same search algorithm changed when implemented on networks with different structures. In the present study, we examined how different algorithms might perform on the same network to determine if certain network features conferred some protection to phonological processing.

In addition to being motivated by network science work from [11,12], the present study was also motivated by psycholinguistic work from [33], among others. The researchers in [33] examined in younger and older adults the speech error known as the tip of the tongue. As described above (to motivate the removal of connections instead of nodes in Study 1), in the tip of the tongue state the speaker might be able to retrieve some information from the lexicon related to the meaning, gender, or syntactic class of a word, but they are not able to completely access the phonological word-form, perhaps retrieving the number of syllables the word has, or the sound with which it starts. It was found that older adults tended to experience more tip of the tongue states than younger adults [33]. They accounted for that difference with the transmission deficit hypothesis in a model known as Node Structure Theory [63].

In Node Structure Theory (NST)—an interactive model of speech production that is a different type of network model than the ones employed in the present studies—priming energy spreads among connected nodes (akin to the way activation spreads in the present network models). Once sufficient priming energy has accumulated in a node, the node is said to be activated, which makes the information associated with that node (e.g., semantic or phonological information) available to awareness. Once sufficient priming has accumulated in the semantic nodes to activate the meaning of a word, priming energy then begins to spread from the semantic node to the phonological nodes associated with that word. Under normal circumstances, sufficient priming accumulates in the phonological nodes associated with that word, thus activating the word-form, and making it available for production.

In the tip of the tongue state, priming spreads from the activated semantic node to the phonological nodes associated with that word. However, not enough priming accumulates to fully activate the phonological nodes, giving the speaker the feeling that they know the word (i.e., semantic information is available), but they can only produce the first sound or syllable of the word, not the whole thing [33].

According to the transmission deficit hypothesis in NST, the connections between nodes weaken with normal aging, resulting in priming being spread less efficiently across the connections between nodes. With priming being transmitted less efficiently with age, it is likely in older adults that the amount of priming that will accumulate will be insufficient to fully activate some nodes, resulting in older adults experiencing the tip of the tongue state (where semantic, but not phonological nodes are activated) more than younger adults, as observed by [33].

In the present study we again used *spreadr* to simulate lexical retrieval for the words from the PNT as we did in Study 2. In the present study, however, we did not use damaged networks as we did in Study 2. Rather, we used the “intact” network from [3], and manipulated the decay parameter (which controls the proportion of activation lost at each time step) to simulate something akin to the transmission deficit hypothesis in NST. For other examples of the decay parameter being manipulated in *spreadr* see [42,43]. By using the decay parameter to manipulate the efficiency with which activation spreads in the intact network we could examine how the structure of the network might “protect” certain nodes despite less efficient processing in the network (perhaps due to normal aging). For other examples of algorithms changing over time in a complex network model see [64,65,66]. Manipulating a parameter in the model related to the efficiency with which activation spreads in the network can also be viewed as another way to model certain types of developmental or acquired disorders in the network approach (as opposed to damaging the structure as in Studies 1 and 2). Although certain age-related changes are normal and typically occur (i.e., they are not related to a developmental or acquired disorder), manipulating the decay parameter in *spreadr* allowed us to model certain (typical) aging effects [33] in the network science approach.

### 4.1. Methods

The lexicon in the present simulation consisted of the 19,340 English words in the phonological network examined in [3]. The stimulus words that were presented to the network to “retrieve from the lexicon” consisted of 165 words from the 175 stimulus words in the Philadelphia Naming Test [44] that were found in the lexicon used in the simulation (see Appendix A).

#### Spreadr

We used the following parameter settings in *spreadr* for the simulation reported here: *initial activation* value of 20 units was used for each stimulus word, *retention* (*r*) = 0.5, *suppress* (*s*) = 0, *time* (*t*) = 5. The settings for these parameters are the same settings as was used in Study 2 (see also [42]).

The present simulation differed from Study 2 in that we manipulated the *decay* (*d*) parameter. *Decay* (*d*) refers to the proportion of activation lost at each time step. This parameter ranges from 0 to 1, and was set to 0.1, 0.3, 0.5, 0.7, and 0.9 in the present simulation.

At the end of five timesteps we documented the activation level of each of the 165 PNT stimulus words in each of the damaged networks. Larger activation values in the simulation correspond to better performance in behavioral tasks (e.g., faster reaction times, more accurate responses, etc., Ref. [41]).

### 4.2. Results

**Figure 6:**
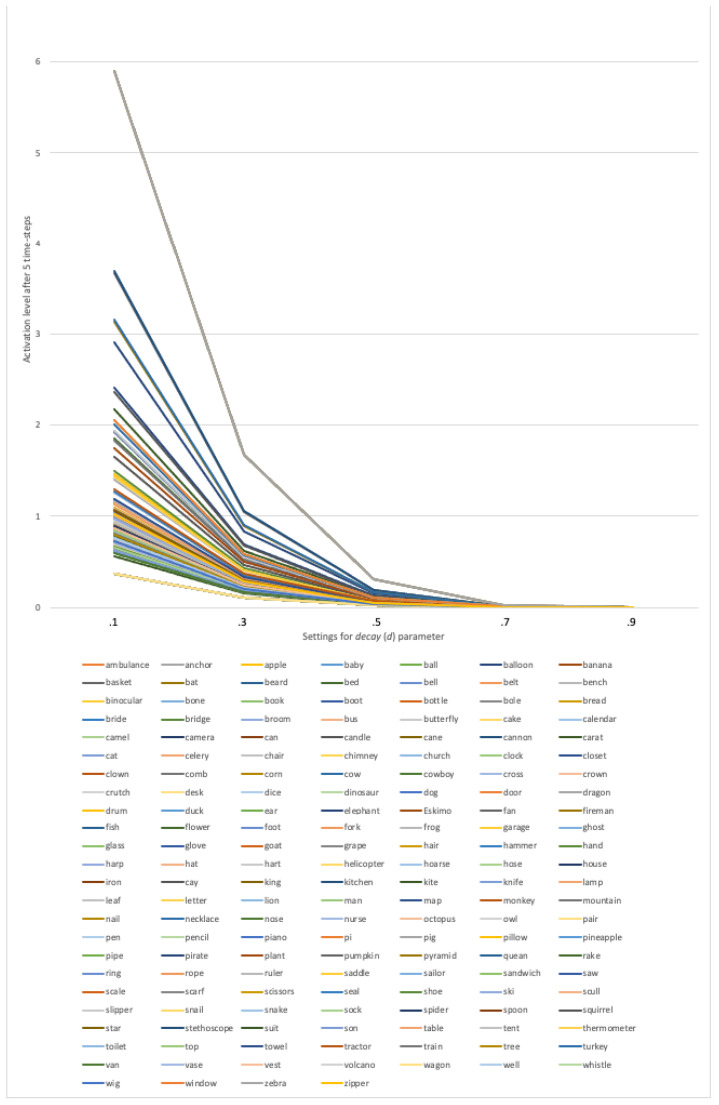
The activation level after five time-steps for each word from the PNT at each setting of the *decay* (*d*) parameter.

In the present simulation we examined how the activation level after five time-steps for each word from the PNT changed at each of the settings for the *decay* parameter in the intact phonological network. Similar to Study 2, we show in Figure 6 the activation value for each word at each setting of the *decay* (*d*) parameter. In comparison to Figure 4, Figure 6 is not as visually dense. Visual inspection of the figure shows that the activation values decrease systematically, and do not intersect at any of the settings for the *decay* parameter. Indeed, Pearson correlations of the activation values for all possible pairs of *decay* settings reveals *r* = +1.0, *p* < 0.001 for all pairings, further indicating that the activation values decrease systematically at each setting of *d*, and do not intersect at any of the settings for the decay parameter.

### 4.3. Discussion of Study 3

The present simulation was motivated by findings from network science and psycholinguistics. Previous network science simulations [11,12] showed that the same algorithm can increase or decrease in efficiency when executed on different networks varying in their structure. In the present case, we examined the “inverse” of those findings; namely, does the structure of the (same) network protect processing when the efficiency of the algorithm is varied?

Varying the efficiency of the algorithm was motivated in part by psycholinguistic work by [33], who found that older adults experienced the tip of the tongue phenomenon more than younger adults. The researchers in [33] accounted for this finding by proposing the transmission deficit hypothesis in the context of the language processing model, Node Structure Theory. With age, priming is transmitted less efficiently between nodes. The less efficient transmission of priming makes it more likely that phonological nodes will not receive sufficient amounts of priming to be activated. If a node is not activated, the information associated with that node will not be available to conscious awareness. The partial priming but not full activation of a node results in the common experience in the tip of the tongue state where one feels that they know a word (i.e., semantic nodes are activated), but the speaker is not able to produce more than the first sound of the word (i.e., phonological nodes do not receive sufficient amounts of priming to activate).

In the present simulation, we manipulated the *decay* (*d*) parameter in *spreadr* to simulate various levels of transmission deficit, as hypothesized by [33] in the psycholinguistic model, Node Structure Theory. The activation level after five time-steps for each word from the PNT at each of the settings for the *decay* parameter in the intact phonological network were then examined. Visual inspection of Figure 6 as well as a statistical analysis of the activation values for each word at each level of *decay* show that the activation values remain parallel at each level of *decay*. That is, the structure of the network “protects” word retrieval despite decreases in processing efficiency; words that are relatively easy to retrieve with efficient transmission of priming remain relatively easy to retrieve with less efficient transmission of priming.

This finding from the present simulation is consistent with the results of a diary study and a laboratory-based experiment of the tip of the tongue phenomenon reported by [33]. They found that the tip of the tongue state tended to occur more for infrequently used words in the language (which are more difficult to retrieve) than commonly used words in the language (which are easier to retrieve), and that this occurred more often for older adults than for younger adults (due to differences in processing efficiency). Thus, the structure of the phonological network can “protect” the retrieval of certain words despite variations in the efficiency with which activation spreads through the network.

## 5. General Discussion

The present studies examined a phonological network that contained nodes representing English words in the mental lexicon, and connections linking words that were phonologically similar [3]. In Study 1 we examined how the continued and random removal of connections altered the overall structure of the phonological network. In Study 2 we examined how the damaged structure of the phonological network influenced processing. Finally, in Study 3, we examined how the structure of the intact network might “protect” lexical retrieval even when a less efficient processing algorithm is used.

The results of Study 1 demonstrated the resilience of the phonological network despite an increasing number of connections being randomly removed from the network. As measured by changes in the average shortest path-length, the phonological network remained relatively well connected until ~90% of the connections were removed, when the average shortest path-length nearly doubled compared to the average shortest path-length in the intact network. The catastrophic shift from “connected” to “disconnected” despite gradually increasing the amount of damage to the system is referred to as a phase transition in the statistical mechanics literature [26].

A central tenet of network science is that the structure of the system influences processing in that system [10]. Given the changes in the structure of the phonological network that we observed in Study 1, we used in Study 2 the *spreadr* program [43] to simulate how the damage to the phonological network might influence lexical processing by comparing the performance of the network model on a set of words to the performance of people with aphasia in a language processing task for the same set of words.

We initially predicted that the damaged phonological networks from Study 1 would result in processing becoming more difficult for the words from the Philadelphia Naming Task (as measured by decreasing amounts of activation remaining in the word nodes after five time-steps). As predicted, we observed a set of words that started out with higher levels of activation remaining in the node after five time-steps, but at some point during the removal of connections, the node became a “lexical hermit” or was stranded on a lexical island of size 2, resulting in lower levels of activation remaining in the node after five time-steps.

Further analysis of the words revealed some additional and unexpected results. Most unexpectedly, we observed a set of words that started out with lower levels of activation remaining in the node after five time-steps, but increasing amounts of damage resulted in higher amounts of activation remaining in the node after five time-steps, indicating that the words had become easier to retrieve with increasing amounts of damage to the network. For these words, damage to the network paradoxically improved lexical processing.

An examination of the network of words immediately connected to these words (referred to as the *ego-network*) revealed that these words initially had many phonological neighbors, which is a situation that typically leads to slower and less accurate spoken word recognition in humans [52] and in computer simulations [41]. Increasing amounts of damage to the network by randomly removing connections resulted in the initially dense phonological neighborhood being “pruned” into a sparse phonological neighborhood (i.e., the words now have fewer competitors), which is a situation that typically leads to faster and more accurate spoken word recognition in humans [52] and in computer simulations [41]. Thus, damage to the phonological network paradoxically improved the processing of some words (i.e., those words that initially had very dense phonological neighborhoods) via the pruning of the phonological neighborhood.

The various ways that damage to the phonological network influences processing (i.e., decrease, increase, or no change in the ease of retrieval) may contribute to the variability in the severity of symptoms often seen among people with aphasia and in other developmental and acquired language disorders. As the results from Studies 1 and 2 suggest, it may not be simply the *amount* of damage to the system that determines the severity of the disorder, but *what* gets damaged as well. For example, damage to words with an initially dense phonological neighborhood may improve lexical retrieval of those words, which could mask an increased difficulty in retrieving other words, resulting in overall behavior being categorized as “mild” in nature despite there being a significant amount of damage to the system.

An important contribution of network analysis is that many conventional measures used in contemporary scientific disciplines are blunt instruments that cannot reveal patterns that are observed with the techniques from network science (as demonstrated by the ego-network analysis in Study 2). Another example of how network analyses reveal patterns that conventional measures cannot used the network approach to represent the syllables of newly learned nonwords, with directed connections linking the first syllable of the nonword to the second syllable of the nonword [67]. Analyses of these syllable networks revealed that children with developmental language disorder (DLD; *aka* specific language impairment) showed a larger inventory of syllable forms, more connections between the forms, and less consistent production patterns compared to typically developing children. These observations would not have been seen had only standard measures of phonetic accuracy been used to analyze the data.

The results of Study 2 also showed that performance on the PNT words in the phonological network with 90% damage correlated with the performance of people with various types of aphasia, demonstrating how damage to the phonological network might influence an acquired language disorder. Recall that we examined aphasia in part because of the availability of data from PWA to use for comparison. We believe, however, that the cognitive network approach coupled with computer simulation can be a powerful way to examine language processing in other developmental or acquired language disorders (e.g., [68]).

Further in Study 2, the post hoc analysis of difficult to retrieve words replicated the findings from [19] regarding the influence of the location of words in the network on lexical retrieval. In the present case, the location of the word in the network influenced naming performance of people with both mild and severe aphasia. Importantly, this factor would not have been revealed had only conventional measures been used, further highlighting the benefit of using network science in the speech, language, and hearing sciences to examine developmental and acquired language disorders.

Furthermore, the results of the post hoc analysis in Study 2 speak to the importance of stimulus selection in anomia interventions for PWA. Treating anomia by focusing on words within the giant component of the network may be of critical importance given their higher-than-expected elicitation of phonologically-related errors. Re-establishing phonological connections of giant component words, using an intact network as the baseline for word location, may result in a more effective anomia treatment. Indeed, there are several phonological anomia treatments that show evidence of improved naming post-intervention, with evidence of generalization to untreated words, Refs. [69,70,71,72]. Continued work focused on anomia treatment stimulus selection, informed by network science and computer simulations, will add to the growing use of cognitive modelling approaches to inform speech, language, and cognitive interventions [73].

Finally, Study 3 examined the resilience of the phonological network in a different way. Rather than damage the network as in Studies 1 and 2, we left the network intact and manipulated the efficiency with which activation could spread through the network. The simulation in Study 3 was partially motivated by previous findings from network science [11,12], as well as by psycholinguistic work on the tip of the tongue phenomenon in younger and older adults by [33]. By manipulating the *decay* parameter in *spreadr* we were able to simulate variations in the efficiency with which activation could spread through the network as proposed in the transmission deficit hypotheses [33]. Manipulating a parameter related to the efficiency with which activation spreads through the network may also be a useful way for modelling other developmental or acquired disorders in the network approach. Despite the variation in the efficiency with which activation could spread through the network, the structure of the network conferred a certain amount of protection on the words. Specifically, words that were relatively easy to retrieve with an efficient transmission of activation through the network remained relatively easy to retrieve when the transmission of activation through the network became less efficient.

Together, the results of Studies 1–3 highlight the value of using network science to study the resilience of the phonological network, and how the resilience of the phonological network might influence various aspects of lexical processing. We recognize that the use of the phonological network limits our ability to capture the effect of semantic information on lexical processing. However, researchers do not need to limit themselves to using networks with only one type of information (e.g., see [74] for an analysis of the semantic network of adolescents with intellectual disability). Future research might be able to overcome that limitation by using *multiplex networks*, which have words in one level connected to each other via their phonological relationship, but connected to each other in another level via their semantic relationship. An additional connection would link, for example, *cat* at the phonological level to *cat* at the semantic level to enable activation at one level to traverse to and influence the other level (e.g., [75]).

Work with multiplex networks containing phonological and semantic levels have already been used to provide insight into word learning [76] and acquired language disorders [26,77]. If one were to include orthographic information about words in a multiplex network (e.g., [78]), then researchers could examine the influence of phonology on reading in typically developing readers as well as in readers with dyslexia or other reading disorders, for example.

Another way in which network analysis has been used is by building “symptom networks” of various pathologies in which nodes represent individual symptoms (as measured by questions on a survey), and connections are placed between symptoms that tend to co-occur. Although this approach is being used increasingly to examine various psychopathologies (e.g., [79]), it can also be used to examine various speech, language, and hearing pathologies to better understand the experience of stuttering [80], and to examine the relationship between a variety of language assessments commonly administered for aphasia diagnosis [81] (for another relevant use of network science see [82]).

Important for the field of neurolinguistics is the application of network analysis to examine the functional and structural connectivity of the brain (for a review see [83,84]). With the neurosciences and the cognitive sciences both using network approaches, perhaps network science can be the *lingua franca* that will connect the mind and brain via a network of networks [85]. In short, network analysis holds much promise for use by clinicians (perhaps to make individualized networks for treatments unique to each person) and by researchers in the speech, language, hearing, cognitive, and neurosciences.

## Figures and Tables

**Table 1:** Network measures of the intact and damaged networks.

	Intact	10% Damage	30% Damage	50% Damage	70% Damage	90% Damage
# of connections	31,267	28,140	21,886	15,632	9378	3124
Giant component size (% of network)	6508 (34%)	6290 (33%)	5870 (30%)	5277 (27%)	4237 (22%)	1767 (9%)
# of isolates (% of network)	10,265 (53%)	10,544 (55%)	11,223 (58%)	12,079 (62%)	13,354 (69%)	15,820 (82%)
# of communities in GC	24	24	27	27	31	38
Modularity value (Q)	0.69	0.69	0.69	0.69	0.71	0.86
Ave. Degree	3.23	2.91	2.26	1.62	0.97	0.32
Ave. Degree in GC	9.11	8.44	6.98	5.46	3.94	2.30
Ave. clustering coefficient in GC	0.32	0.29	0.22	0.16	0.09	0.03
Ave. shortest path length (ASPL) in GC	6.05	6.12	6.29	6.63	7.34	11.93

Note: GC = giant component; Ave. = average (specifically the mean).

**Table 2:** Pearson correlations between simulation activation level in the network with 90% damage and naming accuracy across types of aphasia, and between simulation activation level in the intact network and naming accuracy across types of aphasia.

Aphasia Type	90% Damage	Intact Network
Anomic Aphasia (*n* = 181)	*r* = 0.177 (*p* = 0.022)	*r* = 0.037 (*p* = 0.634)
Broca’s Aphasia (*n* = 85)	*r* = 0.182 (*p* = 0.019)	*r* = −0.020 (*p* = 0.798)
Conduction Aphasia (*n* = 95)	*r* = 0.202 (*p* = 0.009)	*r* = 0.051 (*p* = 0.518)
Wernicke’s Aphasia (*n* = 82)	*r* = 0.169 (*p* = 0.029)	*r* = 0.014 (*p* = 0.854)

## Data Availability

The Moss Aphasia Psycholinguistics Project Database (MAPPD) is available at: http://mrri.org/mappd. The *R* package *spreadr* is available at: https://cran.r-project.org/web/packages/spreadr/.

## Appendix A

The 165 words from the 175 stimulus words in the Philadelphia Naming Test (Roach et al., 1996 [44]) that were found in the lexicon used to construct the phonological network used in the simulations in Studies 2 and 3.

**Words Not Found in the Network**
cheerleaders, eye, fireplace, flashlight, football, microscope, mustache, strawberries, typewriter, waterfall
**Words found in the network**
ambulance, anchor, apple, baby, ball, balloon, banana, basket, bat, beard, bed, bell, belt, bench, binocular (the PNT has binoculars), bone, book, boot, bottle, bowl, bread, bride, bridge, broom, bus, butterfly, cake, calendar, camel, camera, can, candle, cane, cannon, carrot, cat, celery, chair, chimney, church, clock, closet, clown, comb, corn, cow, cowboy, cross, crown, crutch (the PNT has crutches), desk, dice, dinosaur, dog, door, dragon, drum, duck, ear, elephant, Eskimo, fan, fireman, fish, flower, foot, fork, frog, garage, ghost, glass, glove, goat, grape (the PNT has grapes), hair, hammer, hand, harp, hat, heart, helicopter, horse, hose, house, iron, key, king, kitchen, kite, knife, lamp, leaf, letter, lion, man, map, monkey, mountain, nail, necklace, nose, nurse, octopus, owl, pear, pen, pencil, piano, pie, pig, pillow, pineapple, pipe, pirate, plant, pumpkin, pyramid, queen, rake, ring, rope, ruler, saddle, sailor, sandwich, saw, scale, scarf, scissors, seal, shoe, ski (the PNT has skis), skull, slipper (the PNT has slippers), snail, snake, sock, spider, spoon, squirrel, star, stethoscope, suit, sun, table, tent, thermometer, toilet, top, towel, tractor, train, tree, turkey, van, vase, vest, volcano, wagon, well, whistle, wig, window, zebra, zipper
